# Small molecules as theranostic agents in cancer immunology

**DOI:** 10.7150/thno.37218

**Published:** 2019-10-15

**Authors:** Jindian Li, Juno Van Valkenburgh, Xingfang Hong, Peter S. Conti, Xianzhong Zhang, Kai Chen

**Affiliations:** 1 Molecular Imaging Center, Department of Radiology, Keck School of Medicine, University of Southern California, 2250 Alcazar Street, CSC103, Los Angeles, CA 90033, USA.; 2 State Key Laboratory of Molecular Vaccinology and Molecular Diagnostics & Center for Molecular Imaging and Translational Medicine, School of Public Health, Xiamen University, Xiamen 361102, China.; 3 Laboratory of Pathogen Biology, School of Basic Medical Sciences, Dali University, Dali 671000, China.

**Keywords:** small molecules, theranostic agents, cancer immunology, molecular imaging, targeted therapy

## Abstract

With further research into the molecular mechanisms and roles linking immune suppression and restraint of (pre)malignancies, immunotherapies have revolutionized clinical strategies in the treatment of cancer. However, nearly 70% of patients who received immune checkpoint therapeutics showed no response. Complementary and/or synergistic effects may occur when extracellular checkpoint antibody blockades combine with small molecules targeting intracellular signal pathways up/downstream of immune checkpoints or regulating the innate and adaptive immune response. After radiolabeling with radionuclides, small molecules can also be used for estimating treatment efficacy of immune checkpoint blockades. This review not only highlights some significant intracellular pathways and immune-related targets such as the kynurenine pathway, purinergic signaling, the kinase signaling axis, chemokines, etc., but also summarizes some attractive and potentially immunosuppression-related small molecule agents, which may be synergistic with extracellular immune checkpoint blockade. In addition, opportunities for small molecule-based theranostics in cancer immunology will be discussed.

## Introduction

Cancer is still one of the leading causes of morbidity and mortality worldwide. In 2018, 18 million new cancer cases and 9 million cancer-related deaths occurred 1. Recent immuno-oncology therapies have seen significant success by remolding the immune system of patients to treat multiple cancers 2-6.

The mechanism of cancer immunotherapy is based on the blockade of tumor-mediated inhibition of immune responses rather than direct targeting of tumor cells. “Immune checkpoints” means the stimulation or inhibition of receptor-ligand signal axes between tumor cells and immune cells including T cells, dendritic cells (DCs), and macrophages in the tumor microenvironment 7, 8. It wasn't until 1992 when the first immunotherapy drug - PROLEUKIN^®^ (aldesleukin) was approved by the US Food and Drug Administration (FDA), which opened a new era of immunotherapy. Various immune checkpoint-directed antibodies such as anti-cytotoxic-T-lymphocyte-associated protein 4 (anti-CTLA-4), anti-programmed cell death 1 (anti-PD-1), anti-programmed cell death ligand 1 (anti-PD-L1), and anti-CD19 have shown to affect various cancers and are approved by the US FDA 9. In addition, other new and promising drugs for targets such as T-cell immunoglobulin mucin 3 (TIM3), tumor necrosis factor receptor superfamily member 4 (TNFRSF4), and lymphocyte-activation gene 3 (LAG-3) are being investigated in clinical trials, such as NCT02817633 and NCT01303705 (Table 1) 10.

However, new problems have arisen in the course of immunotherapy: 1) nearly 70% of patients who received immune checkpoint therapeutics showed no response or only showed a short-term beneficial effect with recurrence soon afterwards 8; 2) immune checkpoint blockade increases the activity of the immune system and can result in immune-related adverse events such as myocarditis, vasculitis, heart failure, dermatitis, endocrine dysfunction, and even death 11-13; 3) there are primary, adaptive, and acquired resistances to cancer immunotherapies 14; and 4) the disadvantages of antibodies include their long half-life (even multiple weeks) and persistent side effects once injected into the body. Therefore, it is necessary to identify novel immune-related oncology molecular targets and small molecule drugs to expand the treatment range of tumors and/or subtypes of patients, limit adverse events and reduce resistances of immunotherapy.

Recently, combination therapies are widely considered as the most promising oncology treatment strategy. Exploiting intracellular immune-related signal pathways to improve the effect of tumor treatment is an important transition. Intracellular pathways downstream of checkpoint blockade such as the kynurenine pathway, purinergic signaling, and the kinase pathway axis have been explored, and small-molecule-mediated therapeutic agents are being developed, which may show complementary and/or synergistic effects when combined with extracellular checkpoint antibody blockades. Small-molecule drugs possess some advantages over antibodies: 1) the ability go across cellular membranes and other physiological barriers and reach intracellular targets; 2) oral bioavailability; 3) various dosage forms and excellent pharmacokinetic characteristics such as good tumor penetration, efficient delivery into brain tissues, appropriate half-life, and intracellular targets 15; 4) lower manufacturing costs; and 5) diversified strategies for combined therapy by passing into the cytoplasm and interacting with multiple intracellular targets 16, 17. Importantly, kinase-targeted small molecule inhibitors have been established, which are clinically effective and possess appropriate selectivity to avoid or manage clinical toxicities.

This review summarizes the recent use of small-molecule drugs in tumor immunotherapies and immunodiagnostics in (pre)clinical trials, and provides thoughts regarding their future utility both as therapeutic agents and diagnostic tracers. Several comprehensive reviews on small molecules in cancer immunotherapy have been published previously 17-19, which highlighted the small molecule-based immune mechanism, therapeutic compound structures or imaging application in immunity. This review provides a recent update on not only the immune mechanism and therapeutic compounds but also small molecule-based diagnostic radiotracers. Broad applications of small molecules as theranostic agents in cancer immunology are presented and discussed.

## Immune-related targets in tumor microenvironment

Clinical investigators reported that the combination of checkpoint inhibitors with other targeted agents provide multiple points of opportunity for cancer treatments. The various cell types and targets/signal pathways involved in cancer immunity supply prosperous potential targets of intervention for small-molecule-mediated agents, including receptors, extracellular enzymes, and intracellular signal transduction pathways.

**Figure 1:**
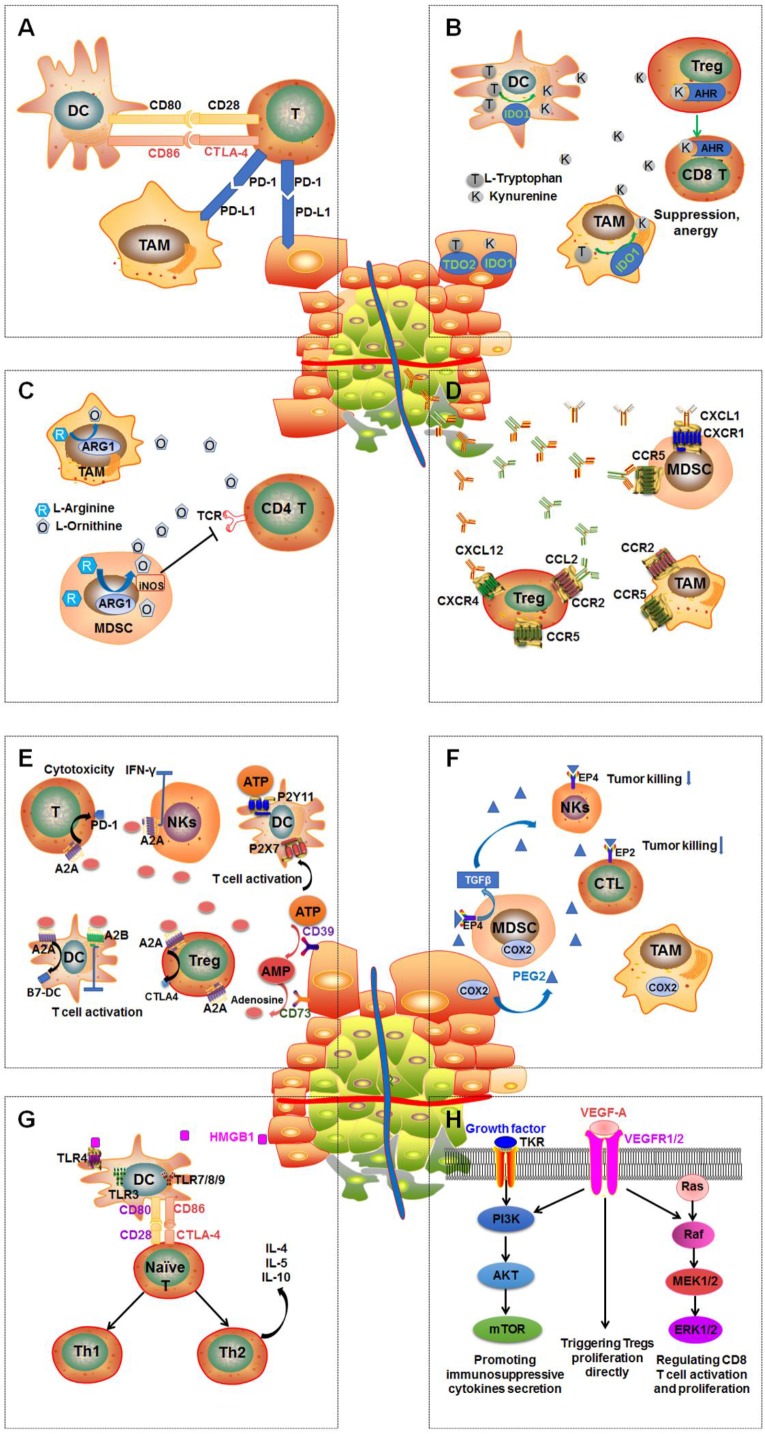
Multiple immunosuppressive mechanisms coexist in tumor microenvironment. **A**: PD-1regulates T-cell activation through binding to its ligand PD-L1. CTLA-4 can stop potentially autoreactive T cells at the initial stage of naive T-cell activation. **B**: Overexpression of DCs and TAMs or IDO and tryptophan 2,3‑dioxygenase (TDO2) in tumor leads to extracellular tryptophan depletion and tryptophan production, subsequently causing defective antigen presentation of DCs and Tregs activations and effector T cell function suppression. **C**: Upregulation of arginase 1 (ARG1) in tumor-associated macrophages (TAMs) and myeloid-derived suppressor cells (MDSCs) results in arginine the depletion, leading to MDSC-mediated immune suppression and impaired CD4^+^ T cell function. **D**: Chemokines CXCL1, CXCL12, CCL2, and CCL5 are secreted by the tumor then spread to the vasculature leading to the recruit and activity of immunosuppressive MDSCs, TAMs and Tregs through interacting with their receptors: CXCR1, CXCR4, CCR2 and CCR5. **E**: ATP is dephosphorylated to adenosine by CD39 and CD73. Extracellular adenosine interacts with their receptors A_2A_R and A_2B_R overexpressed on Tregs, DCs, and T cells to regulate immunosuppressive functions through boosting upregulation of CTLA-4, PD-1 and B7 proteins. **F**: COX2 is overexpressed on tumor cells TAMs and MDSCs to stimulate PGE2 production and then enhance the tumor proliferation and immunosuppressive function of TAMs and MDSCs. In addition, enhancing TGF-β production by MDSCs can inhibit the function of NKs. **G**: Secretion of the high-mobility-group box 1 (HMGB1) protein by dying tumor cells can stimulate the expression of CD80 and CD86 on DCs by binding to TLR4 overexpressed by DCs, which contributes to the differentiation if naïve and/or activated T cells into T helper 1 (Th1) and T helper 2 (Th2). **H**: Activation of PI3K-AKT-mTOR pathway is able to boost expression of immunosuppressive cytokines, chemokines, and checkpoint ligands and recruit regulatory immune cell subsets such as MDSCs and Tregs into tumor. RAS-RAF-MEK-ERK1/2 pathway plays a critical role in CD8 T cell activation, proliferation, and survival by regulating the production of IL-2. The activation of VEGF-A/VEGFR axis enhances PD-1 expression on CD8^+^ T cells leading to the exhaustion of anti-tumor immune cells. In addition, VEGF-A/VEGFR could enhance the pathway of PI3K-AKT-mTOR and RAS-RAF-MEK-ERK1/2.

In general, the tumor microenvironment is quite complex. It is made up of tumor cells, multiple immune cells, lymphovascular cells, and extracellular matrix 20, 21. The interactions between different tumor variants and diverse immune cells either annihilate tumors by activating immune responses or boost immune tolerance and eventually result in tumor proliferation and/or metastasis (Figure 1) 20, 22.

CTLA-4, PD-1 and PD-L1 are considered the most prominent immune pathway checkpoints 23. CTLA-4 and PD-1 are mainly overexpressed by T cells and increase the tolerance of immune cells. PD-L1 is mainly expressed on tumor cells, which binds to PD-1 on immune cells to induce immune suppression. The activation of the PD-1/PD-L1 signal axis also can inhibit proliferation and survival of effector T cells, and secretions of interferon gamma (IFN-γ), interleukin-2 (IL-2), and tumor necrosis factor alpha (TNF-α) 24, 25. CTLA-4 is upregulated on activated T cells. CTLA-4 can resist CD28 activity via binding to CD80 and CD86 with a much higher affinity than that of CD28, subsequently inducing inhibition of T-cell activation. In addition, activated CD8^+^ T cells also overexpress CTLA-4, which counteracts the activity of helper T cells downstream and boosts the regulatory T cells (Tregs) immunosuppressive activity. Therefore, CTLA-4 plays a significant role in the early development of immune tolerance. CTLA-4 inhibitors are able to stimulate activated T-cell activation, subsequently showing antitumor immune response 26.

Tumor-associated macrophages (TAMs) and myeloid-derived suppressor cells (MDSCs) are also able to cause the suppression of immune effectors 27. M2 macrophages present anti-inflammatory and pro-tumorigenic effects such as promoting tumor neovasculature formation, invasion and metastasis. In addition, MDSCs can secrete transforming growth factor beta (TGF-β) and IL-10 to produce direct immunosuppressive effects on T effector cells or induce Tregs generation. Spleen MDSCs are able to downregulate the cell adhesion molecule L-selectin on CD4^+^ and CD8^+^ T cells, resulting in a decrease in the activation and homing of CD8^+^ cells in lymph nodes 28. Cancer-associated fibroblasts (CAFs) can be stimulated by TGF-β and fibroblast growth factor (FGF), thereby promoting tumorigenesis, lymphatic vascularization, and metastasis 29. The function of MDSCs, DCs, macrophages and TAMs can be regulated by indoleamine 2,3-dioxygenase 1 (IDO1), chemokines (CXCRs), arginase 1 (ARG1), or toll-like receptors (TLRs) 17.

Reprogramming of energy metabolism of cancer cells is very important in immunosuppression. Intratumoral hypoxia induces upregulation of hypoxia inducible factor-1α (HIF-1α) through regulating ATP-binding cassette (ABC) transporters. On one hand, accumulated ATP stimulates antitumor immune response via the P2 purinergic receptors (P2XRs or P2YRs) mainly expressed on macrophages, DCs, CD4^+^ T cells, and CD8^+^ T cells. On the other hand, accumulated ATP can be further degraded to adenosine by the catalysis of ectonucleotidases CD39 and CD73 mainly overexpressed on tumor cells, B cells and Tregs 30. Extracellular adenosine mediates immunosuppression by interacting with four subtypes of adenosine receptors, A_1_R (presented on neutrophils and immature DCs), A_2A_R (presented on most immune cells and platelets), A_2B_R (presented on tumor cell macrophages, DCs, and mast cells), and A_3_R (presented on neutrophils and mast cells) 31. In addition, intracellular cyclic AMP (one downstream signaling molecule of ATP) of MDSCs, TAMs, Tregs and tumor cells, is also associated with immunosuppression by COX2 (overexpressed on tumor cells, MDSCs, TAMs, and Tregs), EP2 receptor (presented on cytotoxic T lymphocytes (CTLs) and Tregs), and EP4 receptor (mainly expressed on DCs, natural killer cells (NKs), TH1, and TH17 cells) to regulate MDSCs, Tregs, NKs, and tumor cells 17.

Intracellular signal transduction pathways which are involved in immune resistance have been thoroughly explored. Various kinase signal pathways are the key regulatory factors in the immune system 32. Activin-like kinase 5 (ALK5) can diminish TGF-β signaling leading to activation of CD8^+^ T cells, stimulation of NKs, and generation of CTLs 33. Phosphatidylinositol-3-OH kinase (PI3Kδ) is able to attenuate Tregs function, causing activation of effector T cells 34. Colony stimulating factor 1 (CSF1) stimulates M1 to M2 polarization, and then boosts tumor proliferation and survival 35. Small-molecule drugs, such as vemurafenib and dabrafenib (v-Raf murine sarcoma viral oncogene homolog B1 [BRAF] inhibitors), cobimetinib and trametinib (mitogen-activated extracellular signal-regulated kinase [MEK] inhibitors), and sorafenib, and pazopanib (vascular endothelial growth factor [VEGF] inhibitors) have been approved by the US FDA for the treatment of multiple cancers 36.

## Small molecules as immunotheranostics

Current strategies of immunotherapy aim to reverse immune resistance either by promoting the recognition of tumor-associated antigens or by modulating signals of T cell co-receptors through biological modalities. Multiple clinical trials suggested that small molecule-based approaches of targeted multiple immune-related targets mentioned above show complementary and/or synergistic effect with immune checkpoint inhibitors, which can further promote the response rates of patients and improve survival rates. Nearly 25% of immunotherapy clinical trials combine small molecules as partners for immune checkpoint blockades 37. Therefore, it is necessary to summarize the latest developments of small-molecule-mediated targeting agents as immunotherapies in cancer, and to offer considerations about their utility both as mono-agents and/or in combination with other anti-cancer drugs.

### PD-1/PD-L1 immune checkpoint

**Figure 2:**
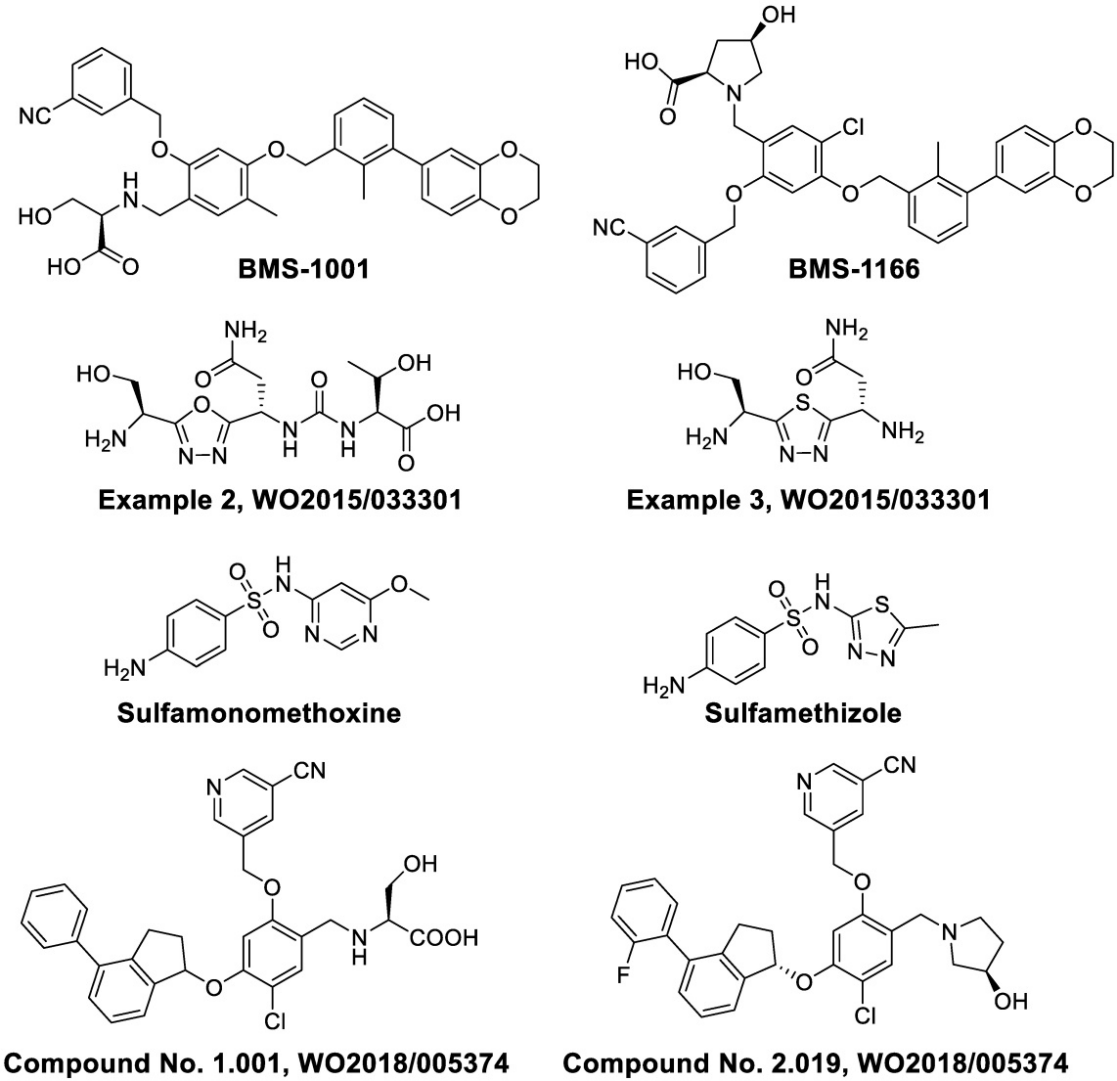
Small-molecule inhibitors of PD-1/PD-L1.

PD-1 is overexpressed by multiple immune cells such as activated T cells, B cells, NKs, DCs, and TAMs, and it is a critical regulator protein for immune inhibition in the innate and adaptive immune systems 38. PD-L1 is overexpressed on various solid tumors and hematological malignancies 39, 40. The PD-1/PD-L1 signal axis inhibits the T cell functions by creating a “molecular shield” in the tumor microenvironment. The interactions of PD-1 and PD-L1 may promote T cell exhaustion, or induce the CD4^+^ and CD8^+^ T cell apoptosis, or enhance immunosuppression of intratumoral Tregs. To date, multiple PD-1/PD-L1 inhibitor antibodies have been effective in some advanced cancer types; however, a remarkable proportion of patients remain resistant to these antibody-based immunotherapies. In order to further expand the response rates of patients to immunotherapies, various small molecule drugs are being explored (Figure 2).

Researchers at Bristol-Myers Squibb (BMS) first synthesized multiple biaryl drugs as PD-1/PD-L1 and CD80/PD-L1 interaction small molecule inhibitors (WO2015/034820). The interaction mechanism may induce the dimerization of PD-L1, subsequently occluding the PD-1 interaction surface 41. The best lead compounds are BMS-1001 and BMS-1166 which can induce dimerization of PD-L1 to exert therapeutic activities 42. BMS-1001 and BMS-1166 can completely restore anti-CD3-mediated T cell activation in nuclear factor of activated T cells (NFAT) luciferase reporter-transfected Jurkat T cells 43. In addition, scientists from Aurigene Discovery Technologies Limited synthesized two compounds with 1,3,4-oxadiazole and 1,3,4-thiadiazole scaffolds (Figure 2, Examples 2 and 3, respectively), which are able to inhibit the PD-1 signaling pathway (WO2011/082400 A3). Sharpe and colleagues synthesized and tested small-molecule PD-1 modulators sulfamonomethoxine and sulfamethizole and their derivatives, which both inhibited the expression of PD-1 in transgenic mouse T cells (WO2011/082400 A3). Most recently, the company Curis Inc. reported compounds CA-170 and CA-327, which not only bind to PD-L1 but also antagonize VISTA or TIM3 binding respectively 44. They can boost T cell proliferation and cytokine secretion. CA-170 is being evaluated in a trial of clinical phase I in humans with advanced solid tumors or lymphomas (NCT02812875). However, their structures have not been disclosed yet.

In 2018, a patent (WO2018/005374 A1) reported by ChemoCentryx Inc. described immunomodulatory inhibitors (Compound No. 1.001 and Compound No. 2.019) that are able to inhibit the PD-1 pathway as shown by ELISA platform-based biochemical interaction assay using human PD-L1.

### Amino acid catabolism

The metabolism of amino acids plays an important role in regulating the innate immune response when diseases occur. Particularly the catabolism of tryptophan and arginine can regulate the immune responses to T cell proliferation and activation. The metabolic pathway of tryptophan catabolized to kynurenine is an essential regulator in maintaining the immunosuppressive microenvironment in many types of cancers. Indoleamine-2,3-dioxygenase (IDO) and TRP-2,3-dioxygenase (TDO) are the key and rate-limiting enzymes in establishing and maintaining immune privilege in tumor immune escape 45. Recruitment of ARG1-expressing MDSCs at a tumor site results in the depletion of L-arginine, which causes reduced proliferation of T-cells and NKs and inhibition of the antitumor immune response. Recruitment of ARG1-overexpressed MDSCs at a tumor site causes L-arginine depletion, which decreases the proliferation of T-cells and NKs and inhibition of the antitumor immune response.

#### Indoleamine-pyrrole 2,3-dioxygenase (IDO)

The IDO family consists of IDO isozymes (IDO1 and IDO2) and tryptophan 2,3‑dioxygenase (TDO2), catalyzing tryptophan to N-formylkynurenine and subsequently to kynurenine and other metabolites. IDOs are overexpressed in macrophages, DCs and various tumor types and contribute to immunosuppression, leading to poor prognosis. The IDO pathway can diminish immune antigen recognition by inducing differentiation and hyper-activation of Tregs and inhibiting immune responses of effector T cells and decreasing DC function. The interaction of kynurenine with aryl hydrocarbon receptor has been verified as a pivotal pathway in immunosuppression functions.

**Figure 3:**
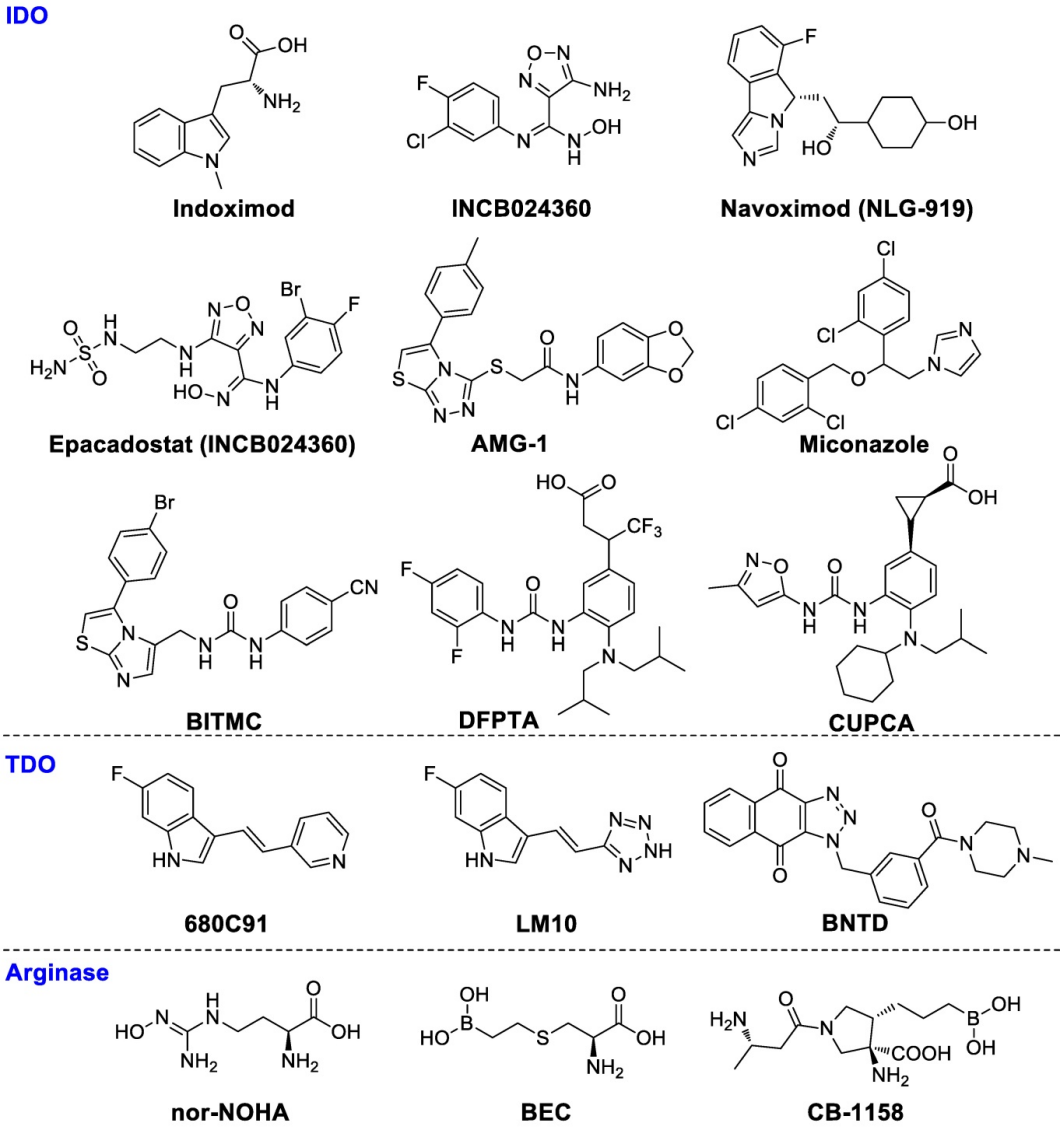
Chemical structures of IDO, TDO, and arginase inhibitors.

D-1MT, or indoximod (Figure 3), the *D*-enantiomer of 1-methyl tryptophan, has been investigated in combination with pembrolizumab in a clinical phase II trial for patients with metastatic melanoma (NCT02073123). Indoximod in combination with a taxane compound is also being investigated in a phase II clinical study for treating patients with metastatic breast cancer (NCT01792050). INCB024360 (Figure 3), or epacadostat, a high selective human IDO1 antagonist, boosts effector T cells and NKs growth, enhances IFN-γ production, increases the amounts of CD86^high^ DCs and diminishes Tregs conversion 46. Although phase III clinical trials (NCT02752074) showed that epacadostat in combination with pembrolizumab for the treatment of melanoma did not reveal superior outcome compared to pembrolizumab alone, the failed trial may have several caveats: 1) it is uncertain as to whether the target was adequately inhibited; 2) a mechanistic rationale for the combination needs to be tested further. Nevertheless, regarding immune-related toxic effects, epacadostat in combination with pembrolizumab therapy seemed to be well tolerated compared with other immunotherapy combinations such as ipilimumab plus pembrolizumab 47. Better rationalized compounds and trial designs will be significant in the future to accurately evaluate medical impact. IDO1 antagonist navoximod (NLG-919/GDC-0919) has also entered a clinical phase I trial for the therapy of advanced solid tumors (NCT02471846). The combination of navoximod and atezolizumab showed acceptable tolerability, safety, and pharmacokinetics for patients with advanced cancer. However, there was no clear evidence of benefit from adding navoximod to atezolizumab although activity was observed. In a CT26 colon carcinoma model with high IDO1 activity, PF-06840003 can reduce over 80% intratumoral kynurenine levels and inhibited tumor growth both in monotherapy and, with an increased efficacy, in combination with a humanized anti-PD-L1 antibody avelumab 48. AMG-1 49, miconazole 50, imidazothiazole derivative BITMC 51, and a 2-aminophenylurea derivatives DFPTA and CUPCA (Figure 3) are some promising lead compounds 52.

#### Tryptophan-2,3-dioxygenase (TDO)

TDO is significantly overexpressed in glioma, lung, breast and colorectal cancer, which is closely related to malignant progression and poor survival 53-58. In glioma, the higher tumor TDO expression is negatively correlated with CD8^+^ immune cell infiltration 53. Salter et al. first reported a new TDO inhibitor 680C91 (Figure 3), which could effectively inhibit TDO activity, but the solubility and bioavailability were poor 59. LM10 is a potent TDO inhibitor with higher solubility and better bioavailability as compared to 680C91. The plasma concentration of LM10 is about 330 times over the 680C91 after seven days of oral administration of 160 mg/kg/day. LM10 demonstrated convincing anti-tumor activity in a preclinical assay showing approximately 57% inhibition of mice with tumor progression compared to normal drinking water (P < 0.001) 54. Wu and coworkers synthesized a highly potent TDO inhibitor BNTD, but further evaluations should be done to verify its therapeutic effects *in vivo* 60. In a word, TDO inhibitors as novel cancer treatments need further investigations.

#### Arginase

The arginine catabolism pathway is a promising approach to reversing immune suppression in the tumor microenvironment 61. MDSC and TAMs both express ARG1, which can decompose the amino acid arginine into ornithine and urea. The consumption of extracellular arginine leads to the CD3ζ chain depletion of T-cell receptors (TCRs), subsequently causing tolerance of T cell responses to antigens. The concentration of ARG1 in MDSCs is elevated in breast cancer and renal cell carcinoma 62, 63. ARG1 inhibition has been shown to prevent lung carcinoma proliferation in mice 64. Small molecule arginase inhibitors including nor-NOHA 65, 66, BEC 67 and CB-1158 are under evaluation.

### Chemokines and chemokine receptors

Chemokines are the pivotal mediators of cancer related chronic inflammation, which modify expression in malignancies, and mediate leukocyte activation and recruitment, angiogenesis, and the proliferation and metastasis of cancer cells. More importantly, the appropriate recruitment of immune cells is orchestrated by the temporal and spatial expression of chemokines and chemokine receptors 68.

#### CXCR family

**Figure 4:**
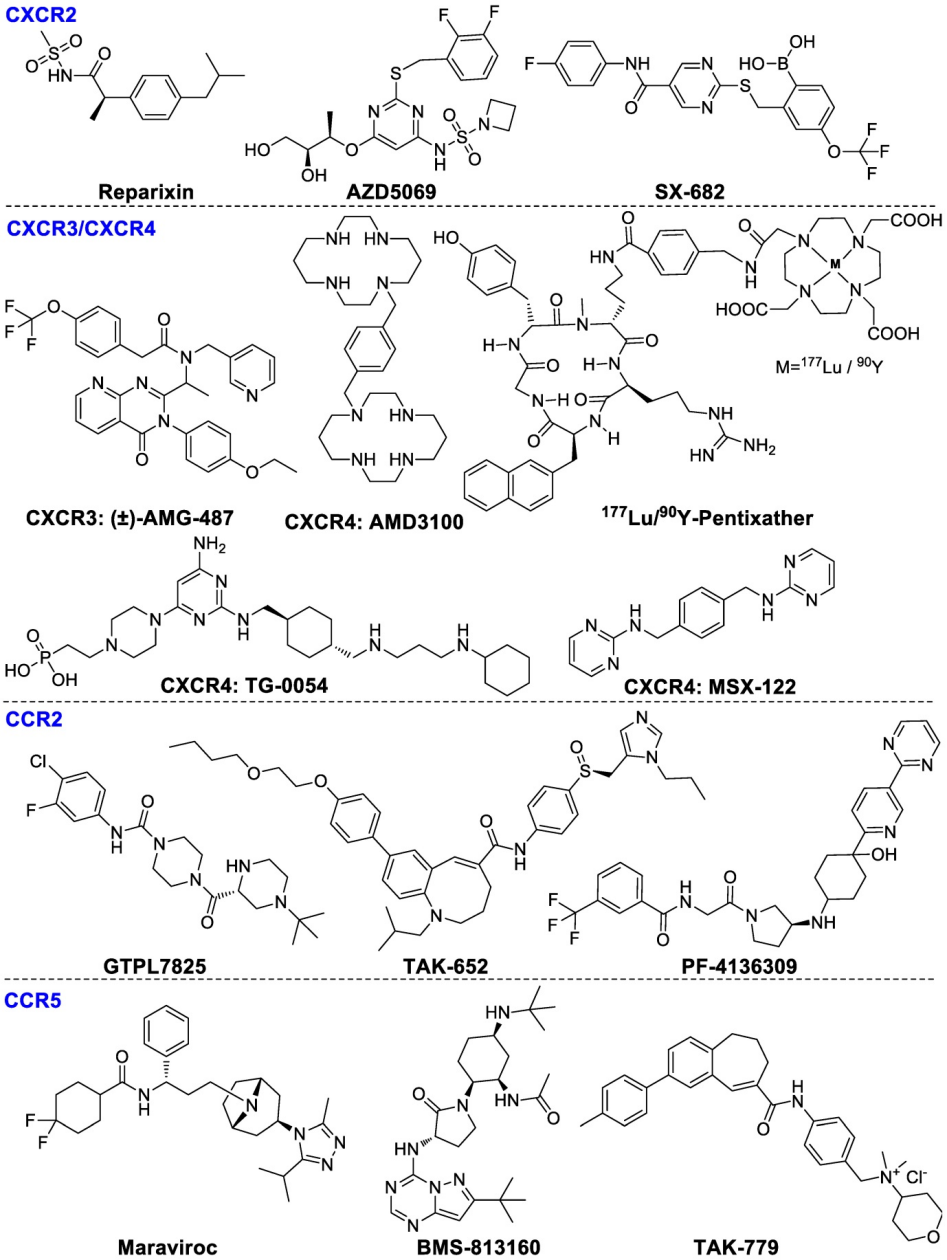
Ligands of chemokine receptors CXCR2, CXCR3, CXCR4, CCR2, and CCR5.

Chemokines, secreted proteins, are critical for lymphoid system development and homing, retention, infiltration and activation of T cells to tumors 69. Chemokines are divided into four main subgroups: CC, XC, CXC, and CX3C. Chemokines are mainly found on the surface of immunocytes, tumor cells and stromal cells. The main immunological function of CXCR2 is to regulate trafficking of neutrophils from the bone marrow to inflammation sites 70. In addition, CXCR2 also modulates MDSCs migrations and mediates local immunosuppression 71. CXCR2-targeted reparixin and PF-04136309 have been investigated in clinical studies for the therapy of breast cancer and pancreatic neoplasms patients respectively 17. The combination AZD5069 (CXCR2 antagonist) with durvalumab (ant-PD-L1) is being investigated in phase Ib/II trials in patients with advanced solid malignancies and metastatic pancreatic ductal adenocarcinoma 72. SX-682 73, a dual CXCR1/2 antagonist, in combination with pembrolizumab is also being investigated in clinical phase I for metastatic melanoma therapy (NCT03161431). CXCR3 is mainly overexpressed by effector CD8^+^ T cells, NKs and TH1 cells. The ligands of CXCR3 are CXC-chemokine ligand 9 (CXCL9) and CXCL10, whose elevated levels are related with enhanced amounts of tumor-infiltrating CD8^+^ T cells, subsequently decreased cancer metastasis and improved survival rates of colon cancer and ovarian cancer patients. CXCR3-targeted AMG487 remarkably decreased metastasis and enhanced host anti-tumor immunity in a 4T1 mammary tumor model 74. The CXCR4-CXCL12 signaling pathway mediates Tregs homing to the bone marrow 75 and plasmacytoid precursor dendritic cells transitioning into tumors 76, regulating metastasis and vascularization of the tumor 77. CXCR4 antagonist plerixafor (AMD3100) combining with anti-PD-1 induced T-cell rapid accumulation among cancer cells and acted synergistically with α-PD-L1 to significantly decrease tumor volume 78. In addition, CXCR4-targeted endoradiotherapy with ^177^Lu- or ^90^Y-pentixather were well-tolerated and exerted anti-myeloma activity even at patients with advanced stage multiple myeloma. Nevertheless, further assessment of toxicity studies and prospectively designed clinical trials is highly warranted 79. Pentixather also can be developed as different diagnostic agents when it is radiolabeled with shorter half-life radionuclides such as ^68^Ga 80, 81. Other CXCR4 inhibitors such as TG‑0054 and MSX-122 are being investigated in clinical studies currently 82, 83. The ligand structures of CXCR family are summarized in Figure 4.

#### CCR family

The CCR2-CCL2 pathway axis is able to induce macrophage migration into the tumor microenvironment and stimulate tumor proliferation and invasion 84. Various CCR2 antagonists have been investigated in clinical studies including GTPL7825, TAK-652 and PF-04136309. PF-4136309 can enhance antitumor effects of the immune system and inhibit tumor proliferation and invasion in patients with pancreatic cancer 85.

CCR5 is primarily expressed by lymphocytes, macrophages and metastatic tumor cells. The upregulation of CCR5 on CD8^+^ T cells, TH1 cells, monocytes and macrophages promotes Tregs infiltration and stimulates progenitor cells differentiation into TAMs and MDSCs 86. Maraviroc was shown to block CCR5 and inhibit tumor metastases in colorectal cancer patients in a clinical phase I evaluation (NCT01736813). In addition, BMS-813160, a dual CCR2/5 antagonist, in combination with nivolumab has been developed for the therapy of patients with colorectal and pancreatic cancers (NCT03184870). TAK-779 is able to block migration of tumor-associated Tregs consequently inhibiting tumor growth specifically in pancreatic adenocarcinoma 87. The ligand structures of CCR family are summarized in Figure 4.

### Purinergic signaling

Hypoxia activates tumor cells to release the pro-inflammatory adenosine triphosphate (ATP), which is subsequently dephosphorylated to immunosuppressive adenosine by CD39 and CD73. The biological actions of adenosine and ATP depend on the activation of purinergic receptors such as P2Xs, P2Ys, CD39, CD73 and adenosine receptors, which are significantly overexpressed on tumor cells and infiltrating immune cells 88.

#### P2 family

**Figure 5:**
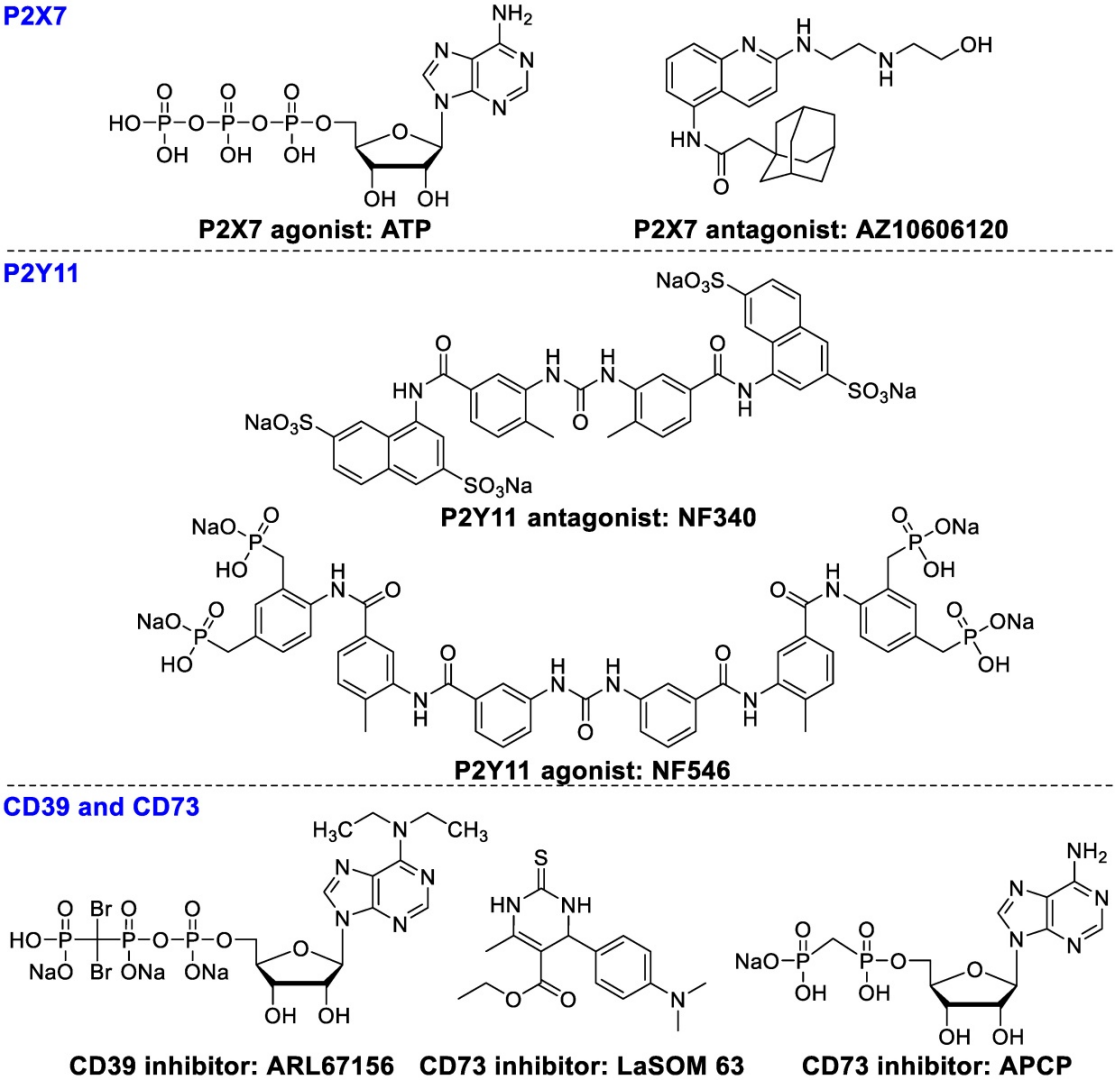
Ligands of P2X7. P2Y11, CD39, and CD73.

P2Xs (ion channel receptors) and P2Ys (G protein-coupled receptors) are overexpressed on various immune cells in the tumor microenvironment. High levels of extracellular ATP can stimulate P2X expression in macrophages and DCs, inducing IL-1β secretion, subsequently enhancing the cytotoxicity of CD8^+^ T cells. Extracellular ATP can also induce apoptosis of Tregs and diminish immunosuppressive activity 89. However, some studies have shown the opposite results, where overexpression of P2Xs promotes tumor growth and survival *in vivo* . AZ10606120 (Figure 5), an antagonist of P2X7, significantly inhibited tumor growth. These contradictory data may result from a slow upgrading of extracellular ATP levels, which causes differences in acute apoptotic response. P2Y11 receptors moderate ATP-induced semi-maturation of monocyte-derived dendritic cells and mediate dendritic cell-based immunotherapy 90. P2Y11 antagonist NF546 stimulated thrombospondin-1 and interleukin 8 (IL-8) release and inhibited lipopolysaccharide-stimulated IL-12 secretion, whereas agonist NF340 reversed these effects 91.

#### CD39 and CD73

CD39 and CD73 play a pivotal role in tumor immunosuppression through converting ATP and ADP to AMP and then to adenosine, resulting in immunosuppression and subsequently the onset and progression of tumor growth 92-94. CD39 is overexpressed on endothelial cells, leukocytes, and B cells 95. CD39 modulates immune and tumor cells to promote tumor growth by catalyzing extracellular ATP or ADP to AMP 96, 97. Subsequently, AMP is hydrolyzed by CD73 into adenosine, which is responsible for immunosuppressive and anti-inflammatory functions of Tregs 93, 98. In addition, T-cell subsets Thpp cells also overexpress CD73 and suppress the CD4^+^ or CD8^+^ T cell proliferation in the presence of exogenous AMP 99. ARL67156 (Figure 5) inhibits the activity of CD39 and partially overwhelms hyporesponsiveness of T cell in some patients with follicular lymphoma 100. LaSOM 63 is able to inhibit the activity of Ecto-5' Nucleotidase/CD73 subsequently causing glioma cell apoptosis 101. APCP, a selective CD73 inhibitor, inhibited tumor proliferation and enhanced efficacy of adoptive T cell therapy 102.

#### Adenosine A2A receptor (A_2A_R) and adenosine A2B receptor (A_2B_R)

After ATP is dephosphorylated to adenosine by CD39 and CD73, the accumulated extracellular adenosine interacts with receptors A_1_R, A_2A_R, A_2B_R and A_3_R which regulate immunosuppressive functions 31, 103. Cyclic AMP (cAMP) is a downstream signaling molecule of adenosine receptors, which is stimulated by A_2A_R and A_2B_R, thereby enhancing immunosuppressive functions 104-106.

A_2A_R is mainly expressed on lymphocytes, NKs, DCs, and T cells. Activation of A_2A_R on T cells markedly inhibits TCR-mediated cytotoxicity and cytokine production, and restrains proliferation of T cells 107. On the other hand, A_2A_R activation can boost Tregs expansion which ultimately enhances immunosuppressive activity 108. CPI-444, a selective A_2A_R inhibitor, was used as a mono-drug or combined with atezolizumab (anti-PD-L1 antibody) for the therapies of patients with advanced non-small cell lung cancer (NSCLC), renal cell carcinoma (RCC), melanoma, and triple negative breast cancer (TNBC) (NCT02655822). The combination of CPI-444 and anti-PD-1 led to a synergistic inhibition of tumor growth (eliminating tumors in 90% of treated mice) and prolonged survival time compared to either agent alone 109. Based on the promising results, Phase 1b clinical study has been initiated (NCT02655822). Co-targeting A_2A_R (PBF‑509, structure not disclosed) with durvalumab is being evaluated in patients with NSCLC (NCT02403193). AZD4635 as a mono-agent or combined with durvalumab (ant-PD-L1) is being investigated for the therapy of patients with advanced solid malignancies, NSCLC, metastatic castrate-resistant prostate carcinoma (mCRPC), and colorectal carcinoma (CC) (NCT02740985), but it has not been completed until now. A_2A_R antagonist preladenant (SCH58261) could enhance NKs activity in mice with B16 melanoma metastasis 110. A fluorinated polyethylene glycol (PEG) derivative of preladenant is confirmed as a promising immunotherapeutic agent 111. Vipadenant and istradefylline are being evaluated in phase II and III studies in Parkinson's disease, which may be promising for treating cancer patients 112.

A_2B_R receptor is the least sensitive of the four adenosine receptors for the requirement of adenosine concentrations to achieve physiological functions. Expression of A_2B_R is enormously increased under hypoxic conditions. Activation of A_2B_R mainly promotes M1 macrophage to M2 macrophage switching, subsequently inhibiting the antitumor T cell activities and promoting angiogenesis in tumors. Under hypoxia, the A_2B_R overexpression on mature DCs can polarize DCs to a Th2-stimulating phenotype. MRS1754, an A_2B_R inhibitor, can enhance the secretion of IL-12p70 and TNF-α and increase the production of Th1 cytokine IFN-c in an mDCs-T-cell co-culture system 113. PBS1115, an A_2B_R-selective antagonist, increases the accumulation of tumor-infiltrating MDSCs *in vivo* 114. ATL801 not only inhibited the growth of 4T1 breast and MB49 bladder tumors but also reduced the metastasis of breast cancer cells, though it significantly increased the concentration of IFN-γ and chemokine CXCL10 115. CVT-6883, a potent selective A_2B_R antagonist, has entered into clinical trials to treat pulmonary inflammation and injury 116. Therefore, CVT-6883 is a very promising candidate drug for tumor immunotherapy.

#### Elevation of cyclic AMP (EP2 and EP4)

**Figure 6:**
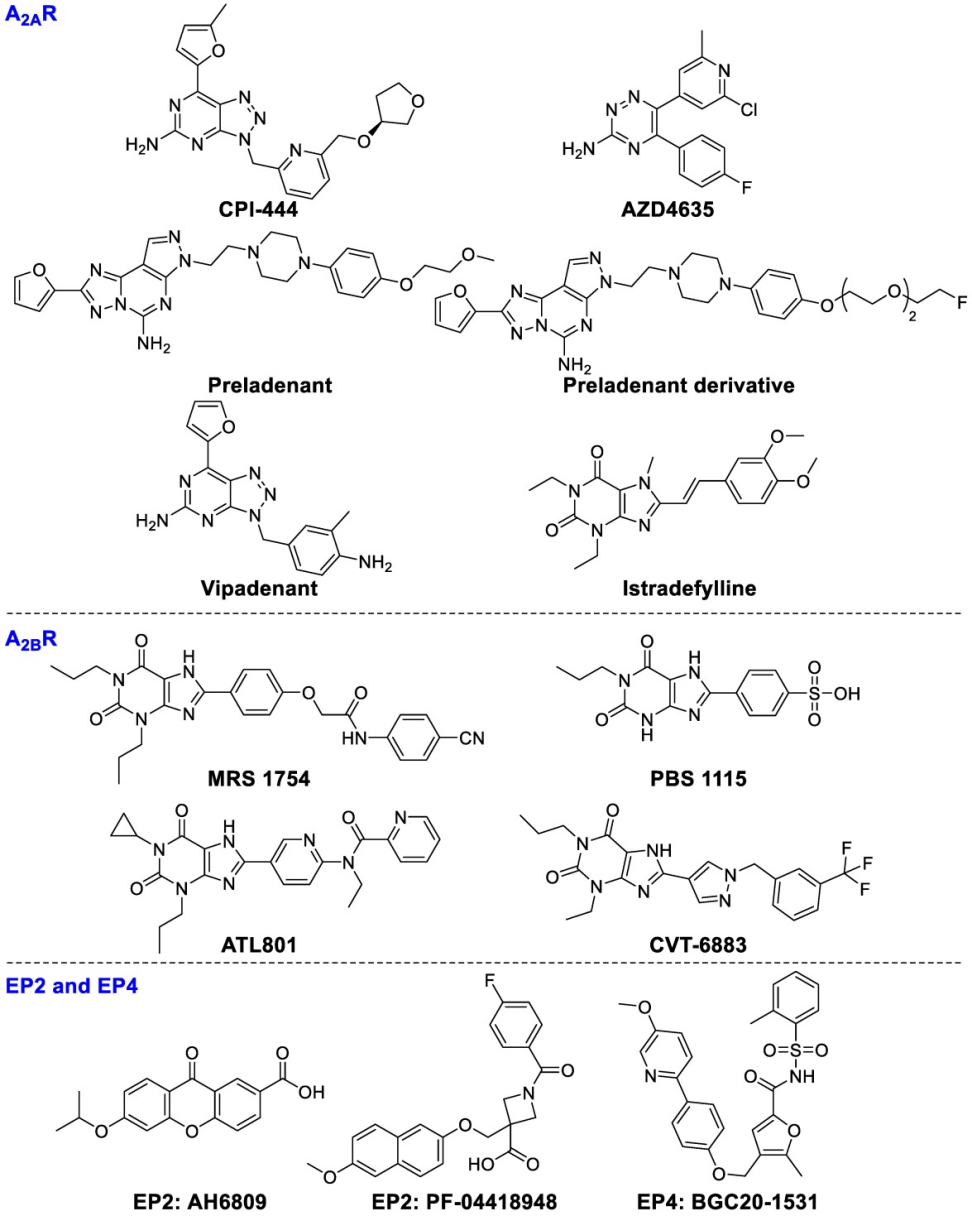
Ligands of A_2A_R, A_2B_R, EP2, and EP4.

The expression of inducible cyclooxygenase (COX2) is correlated with lower survival rates of patients. The metabolites of COX2 are associated with immune tolerance of tumors through stimulating prostaglandin E2 (PGE2) generation to boost tumor proliferation and migration. COX2 upregulation leads to sustained high concentrations of PGE2 117. PGE2 activates its receptors EP2 and EP4 leading to elevation of cAMP levels 118, consequently promoting various immune-suppressive cells activity including Tregs, TAMs, and MDSCs 119-121. AH6809, an EP2 receptor antagonist, can diminish Tregs-mediated immune tolerance 122. PF‑04418948 (EP2 antagonist) 123 and BGC20‑1531 (EP4 antagonist) 124 have been studied in clinical trials for various non-oncology candidates, which may soon expand to anticancer therapy. The chemical structures of ligands are presented in Figure 6.

### Toll-like receptor (TLR) and stimulator of interferon genes protein (STING)

TLRs and STING are regarded as crucial components of the innate immune sensing of tumors. The activation of TLRs and STING in the innate immune system can enhance the secretion of pro-inflammatory cytokines and T-cell recruitment factors subsequently modulate innate immunity, which is able to resist tumor-induced immunosuppression and shows a synergistic effect with present cancer therapies 18.

#### TLR

The TLR family is a critical member of the innate immune system 125. TLRs are primarily expressed by DCs, B cells, neutrophils, monocytes and macrophages, along with the gastrointestinal tract and lungs which are exposed to the external environment. TLRs take part in recognizing pathogen-associated and damage-associated molecular patterns 126. The TLR superfamily contains 13 members; TLR3, TLR7, TLR8 and TLR9 are distributed in the endosomal compartment and others in the cytoplasm. TLR agonists are being evaluated in (pre)clinical studies.

**Figure 7:**
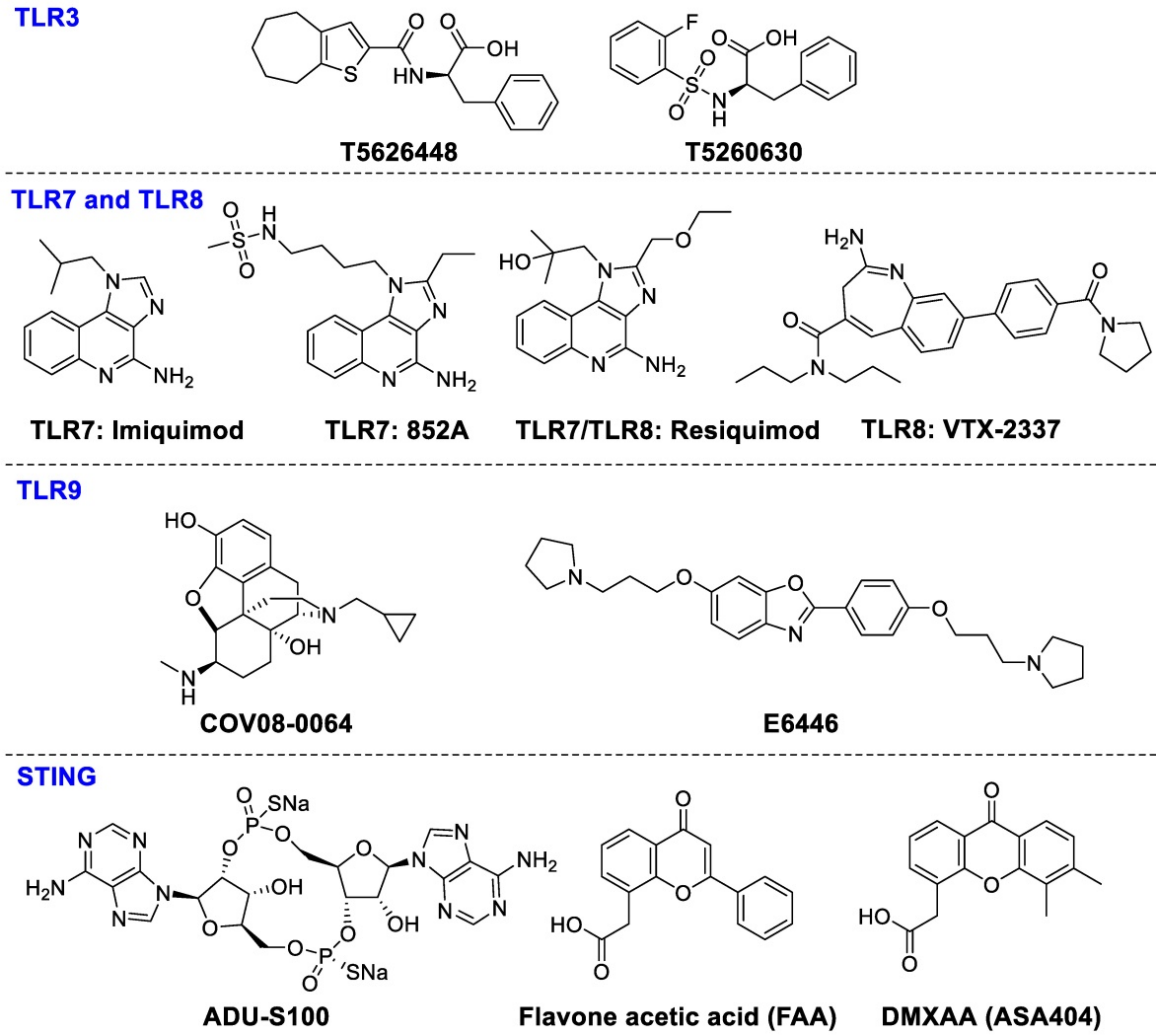
Ligands of TLR3, TLR7, TLR8, TLR9, and STING.

The agonists of TLR3, TLR7, TLR8 and TLR9 make up the majority of preclinical and clinical trials of TLRs. TLR3 signaling is stimulated by dsRNA subsequently causing secretion of pro-inflammatory cytokines and type I interferons such as IL-1, and IL-6, TNF-R to stimulate immune cell activation and recruitment during inflammation or viral infection 127. A series of TLR3-targeted inhibitors, such as T5626448 and T5260630, were evaluated *in vitro* 128. TLR7 and TLR8 are the key targets in the recognition of single-stranded RNA in certain cell types, such as pDC 129. Imiquimod significantly enhanced CD8^+^ T cell accumulation in spleen and draining lymph nodes after administration of DC vaccination 130, 131. Resiquimod, stimulating TLR7 and TLR8, is able to activate immune responses effectively against viral infections and tumors. Resiquimod is in clinical phase II studies for the therapies of viral skin lesions and skin cancer 132. 852A (TLR7 agonist) and VTX-2337 (TLR8 agonist) have been investigated in phase I for treating subjects with advanced solid tumors and lymphoma 133, 134. TLR9 has high affinity for unmethylated and CpG-rich DNA, which is an endosomal receptor for dsDNA in the extracellular compartment. TLR9 agonists COV08-0064 and E6446, can inhibit TLR9-mediated sterile inflammation in acute liver injury and acute pancreatitis models and restrain responses of deleterious inflammation in rodent malaria, respectively 135, 136. Although several trials with TLR modulators are underway, more investigation should be done to achieve more clinical benefits. The chemical structures of TLR ligands are summarized in Figure 7.

#### STING

Transmembrane protein 173 (TMEM173), is expressed in T cells, DCs, and macrophages as well as in various epithelial and endothelial cells. STING activation results in the secretion of cytokines, interferons, and T-cell recruitment factors subsequently modulating innate immunity 137, 138.

STING signals can be activated by cyclic dinucleotides such as cyclic di-GMP and cGAMP, which can induce the expression of interferon-β 139. Recently, ADU-S100 ((*R*, *R*)-S2-CDA) is being evaluated in a clinical phase I study for treating advanced/metastatic solid tumors and lymphomas (NCT02675439). MK-1454 (structure undisclosed) is being studied for treating advanced/metastatic solid tumors or lymphoma in a clinical phase I study (NCT03010176). Flavone acetic acid (FAA) and DMXAA (ASA404) both failed in clinical trials for the therapy of advanced cancer 140. Despite some compounds being in clinical trials for antitumor therapy, administration methods and combinations with other drugs need to be further investigated. The chemical structures of STING ligands are presented in Figure 7.

### Kinase

Kinase signaling pathways can drive many hallmark phenotypes of tumor biology such as metabolism, proliferation, and metastasis. Tumor cells can exert a considerable impact on the microenvironment to inhibit anti-tumor immune responses and escape the pivotal phylactic mechanism. The application of kinase inhibitors can directly inhibit tumor cells, reduce their immunosuppressive influences, and shift the local immunosuppression toward a proinflammatory state, subsequently boosting the activity of the immune activators. Therefore, the combination of kinase inhibitors such as PI3K, MAPK, BRAF, and MEK1/2 inhibitors with immune checkpoint inhibitors is a significant opportunistic proposition to increase the utility of immune modulation in oncology. Various pathways involving kinase inhibitors need to be elucidated to optimize their application in this setting 141.

#### PI3K-AKT-mTOR

Recently, inhibiting the PI3K-AKT-mTOR signal pathway has been evaluated to promote the production of immunosuppressive cytokines 142, 143, the tumor infiltration of MDSCs and Tregs, thereby inhibiting proliferation, migration and survival of tumor cells 144, 145. Thus, it is understandable that PI3K-AKT-mTOR inhibitors plus checkpoint blockade would be effective 146. PI3Kα, PI3Kβ, PI3Kγ and PI3Kδ from the PI3K family are well studied in anti-tumor immunotherapy. PI3Kγ and PI3Kδ are primarily expressed by B and T cells and myeloid lineage cells 17.

##### PI3Kγ

PI3Kγ mainly regulates the innate immune response of myeloid cells by regulating integrin α4β1-dependent macrophage chemotaxis into tumors and suppressing the proliferation and metastasis of tumors 147. TG100-115 and AS605240 could inhibit inflammation, angiogenesis and tumor proliferation in lung carcinoma but without directly affecting tumor cells 147. Infinity (IPI-549) could powerfully inhibit PI3K-γ-mediated neutrophil migration and is presently in clinical phase I studies for the therapy of advanced solid tumors 148. In addition, the combination of IPI-549 with anti-PD-1 treatment enhanced gene expression of anti-tumor immunity and inhibited gene expression of immune-suppressors, thereby hampering tumor growth in primary tumors from human papilloma virus (HPV)^+^ head and neck squamous cell carcinoma 149. These results suggest that PI3Kγ inhibition can synergize with T-cell-targeted immunotherapy to promote anti-tumor immune response.

##### PI3Kδ

**Figure 8:**
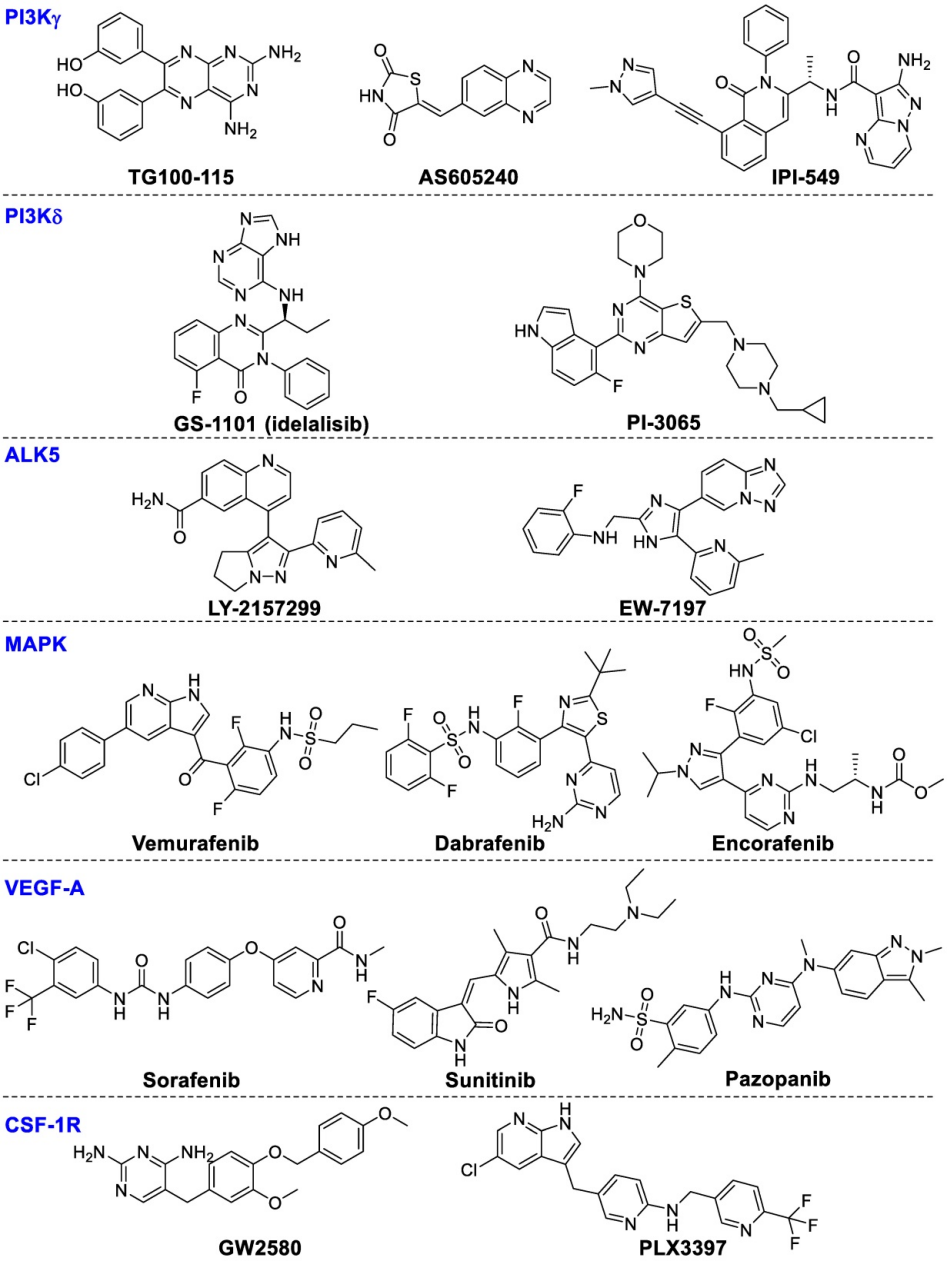
Chemical structures of kinase inhibitors.

PI3Kδ is mainly involved in modulating B-cell proliferation and differentiation. GS‑1101 (idelalisib), a PI3Kδ selective inhibitor, has been approved for treating chronic lymphocytic leukemia. Recently, preclinical results suggest PI3Kδ inhibition in Tregs results in boosting anti-tumor T-cell function and restricting tumor proliferation 34. Idelalisib in combination with pembrolizumab is being investigated for the therapy of chronic lymphocytic leukemia and non-Hodgkin lymphomas (NCT02332980). PI‑3065, a selective PI3Kδ inhibitor, can inhibit tumor proliferation and metastasis in 4T1 breast cancer models. The likely mechanism of PI‑3065's immune regulatory effect may result from enhancing the anti-tumor immune effect though inhibiting Tregs and MDSC function. These results showed PI3K-AKT-mTOR signaling pathways are important targets for the regulation of innate immunity. The chemical structures of the PI3Kγ or PI3Kδ inhibitors are summarized in Figure 8.

#### Activin receptor-like kinase 5 (ALK5)

TGF-β binds to ALK5 and TGF-β receptor type 2 to mediate phosphorylation of SMAD2 and SMAD3. Recently, LY-2157299, a selective ALK5 inhibitor, was able to block TGF-β signaling and inhibit tumor progression in preclinical models. LY-2157299 has entered a Phase I clinical study to evaluate antitumor activity in glioma patients 150. EW-7197 was reported to enhance activation of cytotoxic T lymphocytes thereby inhibiting tumor growth in melanoma-bearing mice 33.

#### Mitogen-activated protein kinase (MAPK)

The MAPK signal axis is activated by various mechanisms, and is a very important target for pathway targeting therapies especially for melanoma metastasis treatment 151, 152. Among the MAPK signaling cascades, the RAS-RAF-MEK-ERK1/2 pathway is very important for CD8 T cell activation, proliferation, and survival, subsequently regulating tumor proliferation and survival 153, 154. Inhibiting the MAPK signaling axis by MEK and B-Raf inhibitors has been an effective therapy for patients with metastatic tumors bearing B-Raf mutations 155. The approved B-Raf inhibitors include vemurafenib and dabrafenib, while encorafenib is being evaluated in multiple phase III trials. In addition, various MEK inhibitors also have been approved such as cobimetinib and trametinib, while binimetinib is presently being studied in various clinical phase III studies. Combinations of MEK inhibitor trametinib with checkpoint inhibitors were more effective than any single drug 156. Clinical evaluation of such combination strategies is underway. It is possible to expand these combinational therapeutic strategies towards other cancer types beyond melanoma.

#### Vascular endothelial growth factor A (VEGF-A)

VEGF-A, a proangiogenic factor produced by malignancies, can enhance the expression of PD-1 on CD8^+^ T cells through an overexpressed VEGF receptor causing the exhaustion of cytotoxic immune cells, which could be reversed by anti-angiogenic agents targeting VEGF-A-VEGFR 157. Some small-molecule VEGF inhibitors including sorafenib, sunitinib and pazopanib have been approved for renal cell cancer. VEGF inhibition enhanced the amounts of tumor-infiltrated effector T-cells and reduced Tregs accumulation in the tumor microenvironment in patients with primary and metastatic renal cell carcinoma 158. VEGF inhibitors in combination with anti-PD-1 or anti-PD-L1 showed positive treatment benefits in patients with renal cell carcinoma or clear-cell metastatic renal cell carcinoma (NCT01472081).

#### CSF-1 receptor (CSF-1R)

CSF-1R signaling is important for recruitment and function of distinct tumor-infiltrating myeloid cells subsets, including TAMs and MDSCs 159. CSF-1R inhibitor GW2580 combined with an anti-VEGFR-2 antibody synergistically inhibits tumor proliferation and hampers tumor angiogenesis 159. PLX3397, a selective CSF-1R inhibitor, is being investigated in clinical trials alone or combined with paclitaxel or checkpoint immunotherapies, or radiation therapy for treating patients with breast cancer, metastatic pancreatic cancer, glioblastoma, and other cancers 17, 160.

## Small molecules for imaging cancer immunotherapy

With the increase in development of personalized-medicine approaches, discoveries of novel immune mechanisms and more selective-targeted drugs approvals, clinicians and researchers need novel methods for exploring the interaction and relationships between tumor cells, the immune system, and immunotherapy agents. It is essential to diagnose whether the patient expresses the related targets before drug administration 161. In addition, it is critical to track the dynamic changes of the targets in immune cells and in tumor cells, to guide clinicians when to switch one drug to another or to determine if a patient no longer needs to receive costly therapy 19. In this way, imaging will provide guidance for physicians to make better decisions on therapeutic regimens and patient follow-up. Several comprehensive reviews on molecular imaging in immunotherapy have been published previously 19, 161-169.

Antibodies can be quickly radiolabeled for imaging via conjugation to a contrast agent or radionuclide. In addition, radiolabeled antibodies keep their naturally high specificity and binding affinity toward their cognate antigens. However, the slow clearance rate and relatively poor penetration into target tissues are drawbacks of radiolabeled-antibody tracers 19. Clinicians must often wait several days before the background signal clears from blood circulation and various non-target tissues. Conversely, radiolabeled small molecules tracers not only possess excellent pharmacokinetic characteristics but also can go across cellular membranes and other physiological barriers and reach intracellular targets. In addition, the manufacturing costs of small molecules tracers are lower than radiolabeled antibodies.

### PD-1/PD-L1 immune checkpoint

**Figure 9:**
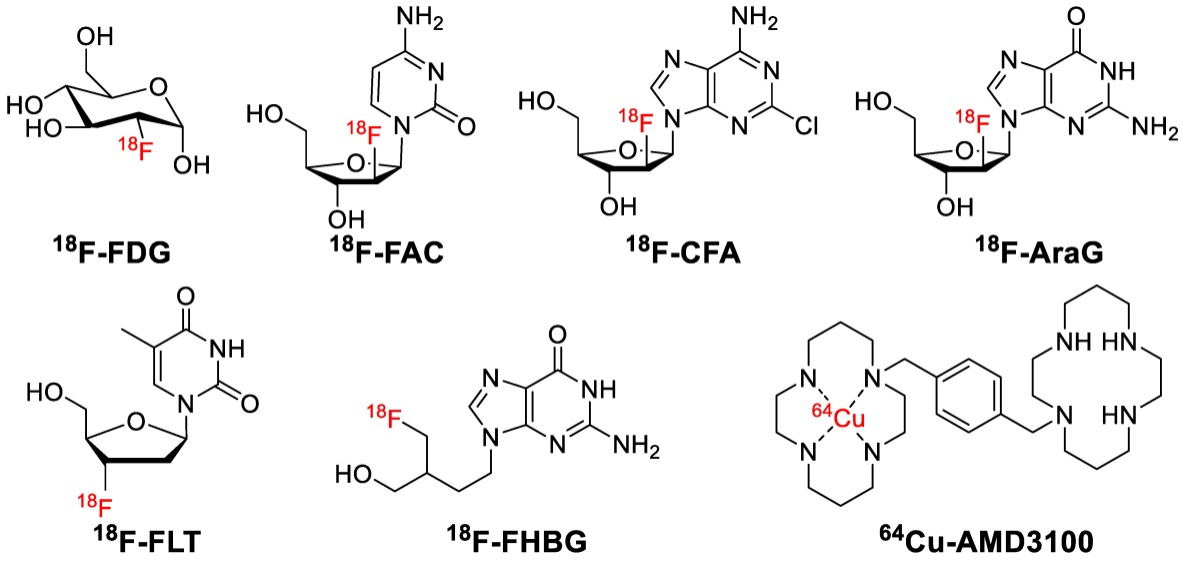
Small molecule-based PET probes for immuno-oncology imaging

**Figure 10:**
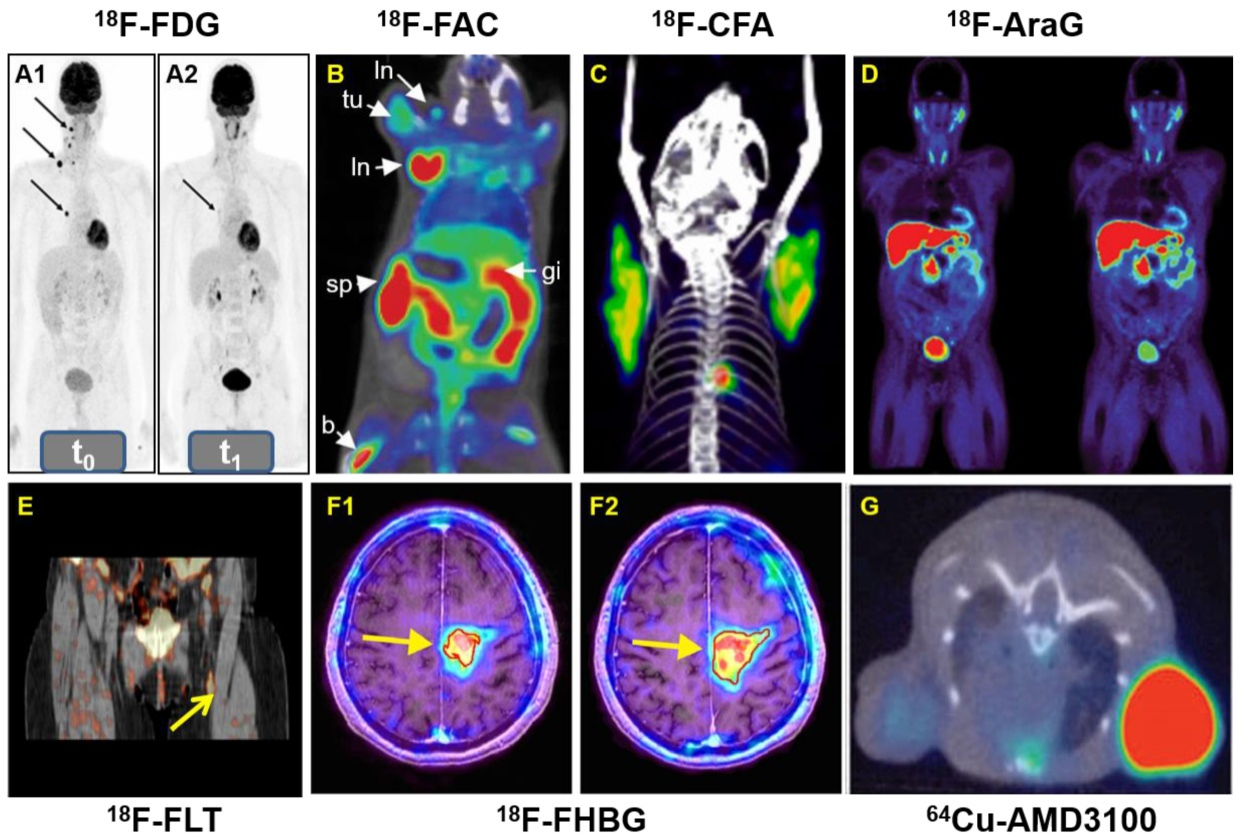
**A**: Whole-body ^18^F-FDG-PET examination at two time points: before PD-1-therapy start (t_0_, base-line) (A1) and two weeks (t_1_, study examination) (A2). **B**: ^18^F-FAC was used to evaluate lymphoid organs and immune activation, tu: tumor; ln: lymph node; sp: spleen; gi: gastrointestinal tract; b: bone. **C**: Detection of immune responses after immunotherapy in glioblastoma using ^18^F-CFA. **D**: Pharmacokinetics of ^18^F-AraG in a healthy human volunteer to prepare to visualize activated T cells in acute graft-versus-host disease. **E**: Early identification of antigen-specific immune responses *in vivo* by^18^F-FLT. **F**: Reporter gene imaging of targeted T-cell immunotherapy in recurrent glioma by ^18^F-FHBG, F1: Pre-immunotherapy using CD8^+^ cytotoxic T lymphocytes (CTLs), F2: Post-immunotherapy using CD8^+^ CTLs. **G**: ^64^Cu-AMD3100-PET/CT for imaging of CXCR4 expression in subcutaneous brain tumor xenografts.

Early detection of therapy response is very pivotal for patients undergoing anti-cancer immunotherapy. ^18^F-labeled fluorodeoxyglucose (^18^F-FDG) (Figure 9) is the most widely used PET probe in nuclear medicine, which is being tested in immunotherapy settings. Chen and co-workers reported that the uptake of ^18^F-FDG has a positive correlation with the expression of both PD-1 and PD-L1 in patients with bladder tumors 170. In addition, Ferdinand and colleagues showed that ^18^F-FDG PET can reliably identify cancer patients who will most benefit from PD-1-therapy as early as two weeks after therapy initiation in stage IV melanoma (Figure 10A) 171. However, ^18^F-FDG used as an immuno-imaging PET tracer needs to be further evaluated in additional clinical trials. It is also expected that the results from ^18^F-FDG can be compared with other specific PD-L1 binding peptide probes, such as ^64^Cu-WL12 172.

### Metabolism of T Cell

PET probes targeting vital metabolic pathways, such as glucose metabolism and nucleotide synthesis and metabolism can potentially be used to monitor the efficacy of immunotherapy involved in innate and adaptive immunity 165.

#### ^18^F-FAC and ^18^F-CFA

In 2008, Radu and co-workers synthesized 1-(2'-deoxy-2'-[^18^F]fluoroarabinofuranosyl) cytosine (^18^F-FAC) to map the deoxyribonucleotide salvage pathway 173. ^18^F-FAC was able to visualize lymphoid organs and was adequately sensitive to localize immune activation in an antitumor immunity mouse model. Additionally, early changes of lymphoid mass in systemic autoimmunity was detected by ^18^F-FAC (Figure 10B), which allowed for the real-time evaluation of immunosuppressive therapy. All the results confirmed ^18^F-FAC can be used for monitoring the process of immune response. However, the clinical application was limited by its rapid catabolism. Kim and colleagues developed an analogue of ^18^F-FAC, ^18^F-Clofarabine (^18^F-CFA), which accumulates in tissues with high dCK expression such as hematopoietic bone marrow and secondary lymphoid organs (Figure 10C) 174. Further studies proved that ^18^F-CFA might be a promising tracer to image the host antitumor immune response against intracranial tumors 175.

#### ^18^F-AraG

9-(β-D-Arabinofuranosyl)guanine (AraG) is an analog of guanosine that has a demonstrable effectiveness for the therapy of T-cell lymphoblastic disease such as recurrent T-cell lymphoblastic leukemia and T-cell lymphoblastic lymphoma. AraG is triphosphorylated by multiple kinases to AraGTP, which preferentially distributes in malignant T-cells. ^18^F-AraG was synthesized and radiolabeled by Ronald et al in 2011, which showed favorable pharmacokinetic properties in healthy humans. PET imaging of ^18^F-AraG may also provide essential information for the early diagnosis of activated T cells in acute graft-versus-host disease (Figure 10D) 176.

#### ^18^F-FLT

Fluorothymidine (FLT), a nucleoside analog, can be quickly absorbed by nucleoside transporters expressed on proliferating cells and then is phosphorylated by the S-phase specific thymidine kinase 1 (TK1), subsequently trapping it within the cells 177. The uptake level of ^18^F-FLT in lymph nodes correlates to the level of antigen-specific IgG antibodies and antigen-specific proliferation of T cells in the blood of patients with metastatic melanoma who received dendritic cell vaccine therapy (Figure 10E) 178.

### Reporter genes of targeted T cells

Reporter gene imaging of engineered T cells is possible when the T cells are transfected with a PET reporter gene which encodes a protein. Reporter gene imaging plays an important role in visualizing their targeting/trafficking, proliferation/expansion, and retention/death using highly sensitive reporter systems, which would provide useful theranostic information 179.

Herpes simplex virus type 1 thymidine kinase (HSV-TK) is a viral nucleoside kinase which is coded by the HSV-TK gene (HSV-tk), whose substrates are thymidine or non-natural nucleosides 180. Preclinical results showed that ^18^F-labeled 9-[4-fluoro-3-(hydroxymethyl)butyl]guanine (^18^F-FHBG) was more sensitive than ^14^C-labeled 1-(2'-deoxy-2'-fluoro-β-*D*-arabinofuranosyl)-5-iodouracil (FIAU) (^14^C-FIAU) in the HSV1-sr39tk system 181. In 2017, Khun and co-workers reported immunotherapy using CD8^+^ cytotoxic T lymphocytes engineered to express both HSV1-TK and IL-13 zetakine chimeric antigen receptor (CAR), which is a promising therapy strategy for patients with recurrent glioma (Figure 10F) 179. Although ^18^F-FHBG PET imaging was safe and facilitated the longitudinal imaging of T cells stably transfected with a PET reporter gene in patients, problems with specificity and viral gene editing in humans may limit their primary application to *ex vivo* immune cell manipulation.

### CXCR4-based immune cells

CXCR4 plays a pivotal role in recruiting immune cells and homing stem cells and progenitor cells 182-184. CXCR4 is overexpressed on multiple human tumor types including esophageal, prostate, ovarian, and renal cell carcinoma, boosting tumor proliferation and metastasis 185, 186. Jacobson and co-workers synthesized CXCR4-specific tracer ^64^Cu-AMD3100, which showed accumulation in CXCR4-expressing organs and tissues 187. Then Nimmagadda and colleagues reported that ^64^Cu-AMD3100 possessed optimized pharmacokinetics and can be applied to decipher graded levels of CXCR4 expression in subcutaneous brain tumor xenografts (Figure 10G) 188. ^64^Cu-AMD3100 is a promising PET tracer for diagnosis of CXCR4 expression.

## Conclusion and perspective

Despite cancer immunotherapy having achieved clinical successes in the past decade, only about 30% of patients have benefited from immunotherapies. There are still many challenges for immuno-theranostics in cancer: 1) additional immune regulatory mechanisms to expand patients' response rates to immunotherapy need to be explored; 2) special immune-competent animal models are required, including transplantable, spontaneous, carcinogen-induced, or genetically engineered humanized malignancies 189; 3) immunotherapeutic effects may also be obstructed by external conditions such as certain bacterial or viral infections, which will modify the immune system 190, 191; 4) reducing or avoiding toxicities caused by general systemic immune activation and developing safer and more effective drugs is essential 192; and 5) more advanced imaging techniques and better characteristic probes need to be developed in order to achieve earlier diagnosis and offer more biological information about multiple cancers 19.

The combination of small-molecule drugs with biologic checkpoint inhibitors is an effective strategy to increase response rates of patients and the efficacy of immunotherapy. It is critical to develop promising imaging technologies and probes to monitor target expression, estimate therapeutic efficacy and potential toxic reactions, and identify who will benefit from immunotherapies. In order to achieve better cancer immune theranostic effect, 1) both intracellular signal pathways down/upstream of immune checkpoints and related therapeutic agents need to be explored; 2) new imaging techniques such as quantum-inspired imaging need to be developed to provide clearer images; 3) mathematic modeling to increasingly derive guiding principles for imaging design and application needs to be optimized; 4) radiomics enabling data to be extracted and applied to improve cancer diagnostic, prognostic, and predictive accuracy should be employed; and 5) more immunoimaging agents need to be developed to keep pace with drug development 19, 193, 194. In addition, immunotherapies can be extended to autoimmune diseases such as graft-versus-host disease 176 and rheumatoid arthritis 195, multiple sclerosis 196 and neurodegenerative diseases 197, 198. Furthermore, artificial intelligence and machine learning will likely improve the efficiency of drug screening and help physicians make better treatment plans.

## Figures and Tables

**Table 1:** Representative drugs approved by the US FDA and other checkpoint inhibitors

(Generic/Brand name)	Target	Mainly indication (Approved time)	Status
Aldesleukin	IL-2 receptor	Metastatic Melanoma (1998.1) Metastatic Renal Cell Carcinoma (mRCC) (1992.5)	Approved
Ipilimumab/ Yervoy	CTLA-4	Metastatic Colorectal Cancer (mCC) (2018.7) Advanced Renal Cell Carcinoma (aRCC) (2018.4) Metastatic Melanoma (2017.7) Late-Stage Melanoma (2011.5)	Approved
Nivolumab/ Opdivo	PD-1	Hepatocellular Carcinoma (HCC) (2017.9) Metastatic Urothelial Carcinoma (mUC) (2017.2) Head and Neck Cancer (HNC) (2016.11) Hodgkin Lymphoma (HL) (2016.5) mRCC (2015.11) Advanced Melanoma (2014.12)	Approved
Pembrolizumab/Keytruda	PD-1	mSCLC (2019.6); Squamous Cell HNC (2019.6); aRCC (2019.4); NSCLC (2019.4); Advanced or Metastatic Merkel Cell Carcinoma (a/mMCC) (2018.12); HCC (2018.11); mNSCLC (2018.10) Primary Mediastinal Large B-Cell Lymphoma (PMBCL) (2018.6) Metastatic Cervical Cancer (mCC) (2018.6) Advanced or Metastatic Gastric Cancer (2017.9) Advanced or Metastatic UC (2017.5); HL (2017.3); mNSCLC (2016.10) Advanced Melanoma (2014.9)	Approved
Cemiplimab/ Libtayo	PD-1	Squamous Cell Carcinoma (2018.9)	Approved
toripalimab	PD-1	Advanced or Metastatic Melanoma	Phase 2
Atezolizumab/ Tecentriq	PD-L1	Extensive-Stage SCLC (2019.3) Metastatic Triple-Negative Breast Cancer (mTNBC) (2019.3.) Metastatic Non-Squamous NSCLC (2018.12) Advanced Bladder Cancer (2017.4) Metastatic Lung Cancer (2016.10) Urothelial Carcinoma(2016.5)	Approved
Avelumab/ Bavencio	PD-L1	Advanced Renal Cell Carcinoma (2019.5) Urothelial Carcinoma (2017.5) Merkel Cell Carcinoma (2017.3)	Approved
Durvalumab/ Imfinzi	PD-L1	Non-Small Cell Lung Cancer (2018.2) Urothelial Carcinoma (2017.3)	Approved
MEDI4736	PD-L1	NSCLC	Phase 3a
Avelumab	PD-L1	Ovarian Cancer	Phase 2
Tisagenlecleucel/Kymriah	CD19	Large B-Cell Lymphoma (2018.5) Acute Lymphoblastic Leukemia (2017.8)	Approved
Axicabtagene ciloleucel/ Yescarta	CD19	Large B-Cell Lymphoma (2017.10)	Approved
Cyclophosphamide	CD19	Lymphocytic Leukemia	Phase 1
Sym023	TIM-3	Metastatic Cancer; Solid Tumor; Lymphoma	Phase 1
TSR-022	TIM-3	Advanced or Metastatic Solid Tumors	Phase 1
MEDI6469	TNFRSF4	Head and Neck Cancer; Progressive Metastatic Prostate Cancer	Phase 1
KHK4083	TNFRSF4	B-Cell Non-Hodgkin Lymphoma	Phase 2
PF-04518600	TNFRSF4	Metastatic Renal Cell Cancer	Phase 2
Sym022	LAG-3	Advanced Solid Tumor Malignancies or Lymphomas	Phase 1
BMS 986016	LAG-3	Gliosarcoma and Recurrent Brain Neoplasm	Phase 1
